# Capacity building in dementia research: insights from the World Young Leaders in Dementia

**DOI:** 10.1002/alz.70667

**Published:** 2025-12-17

**Authors:** Florentina Morello‐Garcia, Nicolas Corvalan, Jorge Llibre‐Guerra, Micaela Arruabarrena, María Florencia Clarens, Greta Keller, Loana De Los Santos, Maria Eugenia Martin, Cristiano Schaffer Aguzzoli, Ricardo Allegri, Livia Amaral, Carolina Ardohain Cristalli, Bruna Bellaver, Merci Ngozi Best, Madeleine Bloomquist, Kevin Chen, Neha Dubey, Cynthia Felix, Diego Fernandez Slezak, Igor Camargo Fontana, Indira Garcia‐Cordero, Micaela Anahi Hernandez, Ozama Ismail, Florence Johnson, Arshia Khan, Suelyn Koerich, Maria Celeste Lopez Moreno, Pamela Lukasewicz Ferreira, Nahuel Magrath Guimet, Markley Oliveira Junior, Tharick A. Pascoal, Guilherme Povala, Andreia Rocha, Matheus Scarpatto Rodrigues, Marina Scop Medeiros, Emma Patrice Ruppert, Kaitlin Seibert, Claire Sexton, Carolina Soares, Ezequiel Surace, Hannah Wilks, Eduardo Zimmer, Lucia Crivelli

**Affiliations:** ^1^ Institute of Neurosciences (INEU) Fleni‐CONICET, Ciudad Autónoma de Buenos Aires Buenos Aires Argentina; ^2^ Department of Cognitive Neurology Fleni, Ciudad Autónoma de Buenos Aires Buenos Aires Argentina; ^3^ Washington University in St. Louis St. Louis Missouri USA; ^4^ Brain Institute of Pontifical Catholic University of Rio Grande do Sul Porto Alegre RS Brazil; ^5^ Department of Psychiatry University of Pittsburgh Pittsburgh Pennsylvania USA; ^6^ Department of Neurology School of Medicine University of Michigan Ann Arbor Michigan USA; ^7^ Department of Neurosurgery School of Medicine University of Michigan Ann Arbor Michigan USA; ^8^ Department of Applied Psychology University of Calcutta Kolkata India; ^9^ Departamento de Computación Facultad de Ciencias Exactas y Naturales Universidad de Buenos Aires (UBA), Pabellón Cero+Infinito, Ciudad Universitaria, Ciudad Autónoma de Buenos Aires Buenos Aires Argentina; ^10^ Instituto de Investigación en Ciencias de la Computación (ICC) CONICET‐UBA, Pabellón Cero+Infinito, Ciudad Universitaria, Ciudad Autónoma de Buenos Aires Buenos Aires Argentina; ^11^ Alzheimer's Association Chicago Illinois USA; ^12^ Tanz Centre for Research in Neurodegenerative Diseases University of Toronto Toronto Ontario Canada; ^13^ Cognitive Neuroscience Center Universidad de San Andrés Victoria Buenos Aires Argentina; ^14^ School of Nursing University of Michigan Ann Arbor Michigan USA; ^15^ University of Minnesota Duluth Duluth Minnesota USA; ^16^ The University of Texas Health Science Center at Houston, McGovern Medical School Houston Texas USA; ^17^ Instituto de Psicología Básica Aplicada y Tecnología (IPSIBAT), Universidad Nacional de Mar del Plata (UNMdP) – CONICET, Mar del Plata Buenos Aires Argentina; ^18^ Department of Neurology University of Chicago Medicine Chicago Illinois USA; ^19^ Laboratory of Neurodegenerative Diseases Institute of Neurosciences (INEU) Fleni‐CONICET, Ciudad Autónoma de Buenos Aires Buenos Aires Argentina; ^20^ Graduate Program in Biological Sciences: Biochemistry (PPGBioq) and Pharmacology and Therapeutics (PPGFT), Department of Pharmacology Universidade Federal do Rio Grande do Sul Porto Alegre RS Brazil; ^21^ Department of Pharmacology & Therapeutics McGill University McIntyre Medical Sciences Building Montreal Quebec Canada

**Keywords:** capacity building, dementia, early career researchers, leadership, low‐ and middle‐income countries, scientific community, World Young Leaders in Dementia

## Abstract

Early‐career researchers from low‐ and middle‐income countries face systemic barriers to professional development and leadership growth. This article presents results from an initiative led by the World Young Leaders in Dementia (WYLD), including a leadership‐focused session at the Alzheimer's Association International Conference 2024 and a global survey completed by 130 dementia researchers from 17 countries. The survey explored five capacity‐building domains critical for leadership development. Over half of the survey respondents stated that scientific research in their country was not prioritized in public policy. Additionally, only 39% report holding full‐time academic positions. The most cited challenges included lack of funding sources, training opportunities, and physical workspace. These findings highlight the urgent need to invest in research, training, and infrastructure to support future scientific leaders. As dementia incidence rises, prioritizing capacity building is essential to ensure global equity in research.

**Highlights:**

Early‐career dementia researchers face major barriers, especially in LMICs.

A networking session and a global survey explored capacity‐building needs in dementia research.

Key obstacles: lack of funding, training, workspace, and protected research time.

Leadership development is a critical component of sustainable research capacity.

## 1 BACKGROUND

Dementia has become one of the greatest global health challenges. Global projections estimate that 152.8 million people will be living with dementia by 2050.^1^ Meanwhile, the scientific community faces profound disparities driven by systemic inequalities and underinvestment, particularly in low‐ and middle‐income countries (LMICs),^2^, ^3^, ^4^, ^5^, ^6^ which are home to two‐thirds of the population worldwide living with dementia.^5^ Without equitable investment in research, infrastructure, and training, the development of the new generation of scientific leaders capable of translating dementia research into real‐world solutions is severely hindered.^7^ To overcome these challenges, capacity‐building initiatives must be strategically designed to close these critical gaps.

Capacity building is defined as the process of developing knowledge, skills, structures, systems, leadership, and social relationships that empower individuals, organizations, and communities to effectively address their needs and challenges.^8^, ^9^, ^10^, ^11^ This concept functions both as an instrumental means to achieve specific goals and as a constitutive end in fostering sustainable development and social equity. In the scientific domain, capacity building can be understood as the development of structures and systems that enable effective research and support the implementation of evidence‐based public policies.

Leadership development is a fundamental element of capacity building. In the scientific domain, this entails not only developing human resources but also fostering leadership that (1) bridges gaps between national and international research groups and (2) promotes the acquisition of essential technical skills, including, but not limited to, clinical trial design, advanced data analysis, statistical programming, neuroimaging processing, genetic data analysis, and the use of digital tools for data management, integration, and interpretation across diverse biomedical domains. In this context, there is a growing consensus on the urgent need to prioritize career development to ensure the sustainability of dementia research globally.^12^ However, such initiatives remain the exception rather than the norm, leaving many researchers, especially those from LMICs,^2^, ^3^, ^4^, ^5^, ^6^ struggling with limited resources, scarce training opportunities, and restricted access to global scientific networks. Additionally, few countries explicitly recognize early‐career researchers in their national dementia plans.^13^ The value of bright, young scientists with novel skillsets is often overlooked, and the support they receive remains insufficient, despite their crucial role in the future of dementia research.

In this context, this article aims to identify the main obstacles faced by dementia researchers and explore key characteristics of leadership in the field. To achieve this, we present (1) insights derived from an “Intermission” networking event hosted by the International Society to Advance Alzheimer's Research and Treatment (ISTAART) and the World Young Leaders in Dementia (WYLD) at the Alzheimer's Association International Conference (AAIC) 2024 and (2) results from a global survey distributed to dementia researchers that focused on key capacity‐building domains, exploring barriers and access to opportunities in their respective fields.

## 2 METHODS

### 2.1 ISTAART intermission networking event at the AAIC

The objective of the ISTAART Intermission titled “A Pathway to Empower Leadership in Dementia” was to promote the exchange of ideas and experiences among young professionals regarding the strengths and leadership qualities needed in dementia research, aiming to identify strategies to enhance these qualities and foster leadership in their region, as well as the barriers and opportunities present in this field. It was organized by WYLD, an international network of young professionals working in an interdisciplinary manner to address the challenges of dementia.

A team of WYLD professionals designed the activities of the ISTAART Intermission, anticipating the participation of approximately 30 people. A focus group was conducted with members of the Neuropsychology Department at Fleni (Buenos Aires, Argentina), and their feedback was used to refine and finalize the activity framework.

The final activity plan included (1) a brief introduction to WYLD as an organization and the importance of connecting young researchers in the field of dementia, (2) development of the overall objective of the ISTAART Intermission, (3) first activity: “icebreaker,” (4) second activity: “building together,” (5) presentation of the work done, and (6) closing.

The activity was promoted through WYLD's and the ISTAART Intermission organizers' social media channels. WYLD also hosted a booth at the conference, where attendees could learn more about the organization and were encouraged to participate in the session, which was open to all conference attendees.

### 2.2 Survey design

A comprehensive survey was developed to investigate the primary challenges encountered by dementia researchers and to explore needs and opportunities related to leadership development in the field. The survey was structured around five key domains of the concept of capacity building: (1) educational training, (2) funding opportunities, (3) infrastructure, (4) external collaborations, and (5) community engagement.^14^ A focus group was conducted with members of the Argentine WYLD team to support the development and refinement of the survey items. Additionally, a pilot version of the survey was tested during the ISTAART Intermission session at AAIC 2024 with the dementia professionals who participated. Feedback and insights gathered from this pilot, along with the outcomes of the interactive activities held during the session, informed the final version of the questionnaire. The questions from the final version of the survey can be found in Appendix A.

The survey aimed to gather insights from a diverse audience with varying academic career trajectories, and it was disseminated through multiple channels, including the WYLD membership list and collaborations with dementia‐related organizations worldwide. The only eligibility criterion for participating in the survey was self‐identification as a researcher in the field of dementia. There were no restrictions based on professional background (e.g., neurologists, neuropsychologists, biologists, data scientists) or career stage (e.g., students, PhDs, postdocs, professors).

This project was approved by the Fleni research ethics committee under registration code 14195.

### 2.3 Data management

Survey data were analyzed using percentage distributions for categorical variables. Data transformations and visualizations were performed using R version 4.3.2 utilizing the ggplot and dplyr libraries.

## 3 RESULTS

### 3.1 The ISTAART intermission experience

A total of 45 professionals attended the ISTAART Intermission event. These researchers are based in Argentina, Brazil, India, Spain, and the United States. They were divided into four groups, each seated at a round table to facilitate discussion and interaction.

For the first activity, “icebreaker,” images of various objects were placed face down on a table. Working in pairs, participants were asked to randomly select one of the cards and identify one positive and one negative trait that the object could symbolize in the context of leadership within dementia research. For example, a megaphone might represent a leader's ability to communicate effectively and the imposition of ideas without considering others’ input. Each pair then shared their insights with the rest of their table group.

Participants identified several positive leadership traits in their responses. The flashlight symbolized qualities such as being discovery‐oriented, having the ability to focus, and highlighting the strengths of others. The compass represented leadership by guiding, inspiring confidence in the plan, and making decisive choices. Meanwhile, the shield with swords conveyed attributes of demonstrating strength, showing bravery, offering protection, and fostering collective efforts to combat dementia.

On the other hand, participants also associated certain negative traits with these symbols. The flashlight was criticized for not allowing other team members to shine. The compass was seen as ineffective when there is no clear goal and was perceived as inflexible in exploring different paths. The shield with swords was linked to negative connotations of using force and authority to solve problems and a lack of communication.

For the second activity, “building together,” participants explored leadership traits in dementia research through a creative group task. Each team selected three symbolic objects from the previous activity, summarized their relevance, and built a physical model using blocks and figurines. They were encouraged to be imaginative and creative, incorporating symbolic elements into their structures to visually convey the leadership characteristics discussed and how these traits might manifest within dementia research.

Finally, to conclude the activity, each group presented their cardboard summary to the larger group audience, explaining the rationale behind their card selections and the leadership concepts they had discussed. Then they introduced their constructed models, describing how each element of the structure symbolized specific leadership qualities and how these aligned with the challenges and dynamics of dementia research.

The reflections shared during the ISTAART Intermission provided valuable insights into how researchers conceptualize leadership in the dementia field. Through symbolic metaphors, participants expressed not only aspirational qualities such as clarity, direction, and protection, but also concerns about hierarchical rigidity, lack of collaboration, and top‐down communication styles. These representations, together with feedback from a pilot version of the global survey (completed by participants at the end of the session via QR code), were used to refine the final version of the questionnaire, whose results are presented in the next section.

### 3.2 Capacity‐building survey results

#### 3.2.1 Survey sociodemographics

**FIGURE 1:**
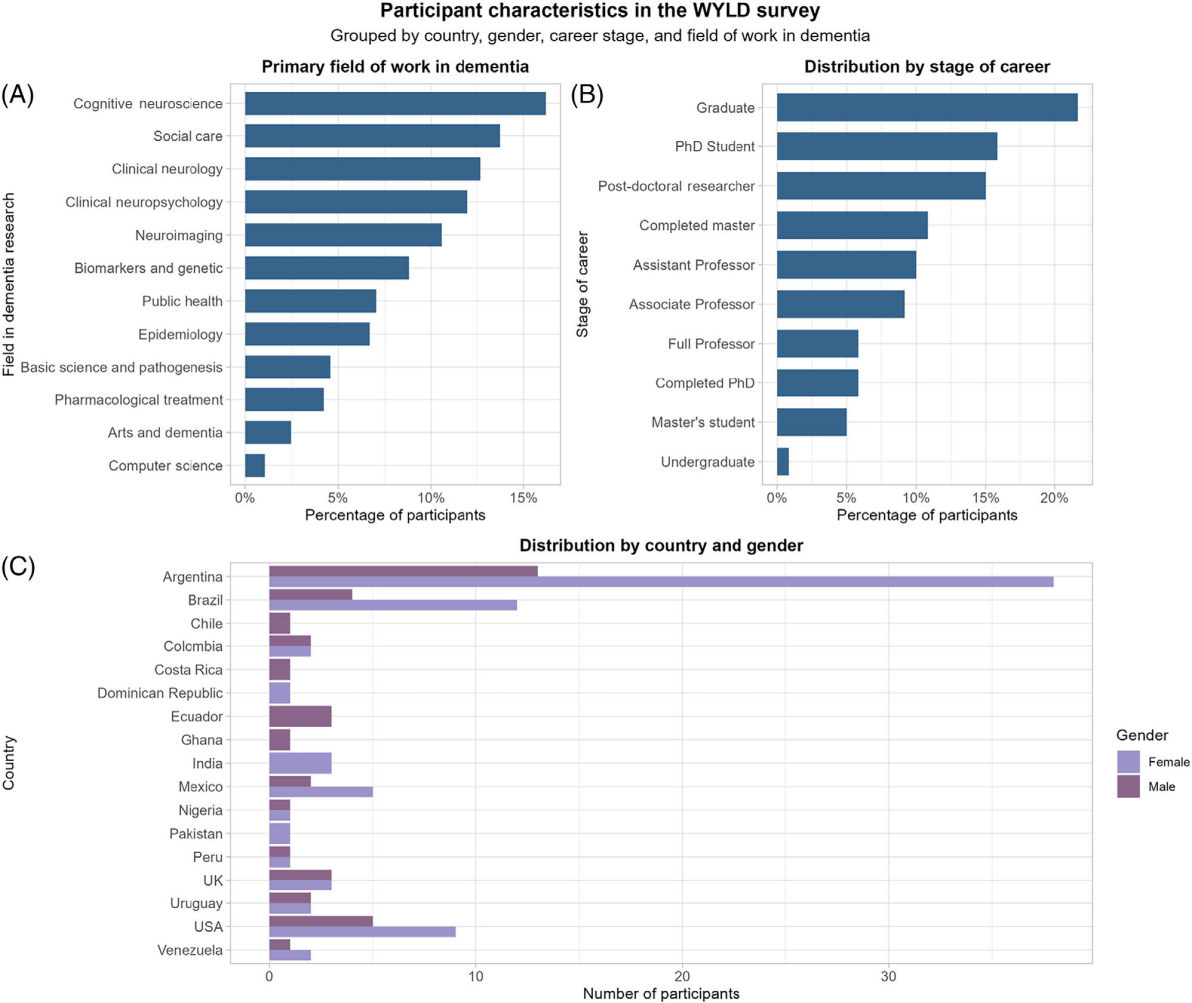
Sociodemographic data from respondents (*n* = 130). The figure summarizes participant distribution by primary field of work in dementia (A), career stage (B), and country and gender (C). Panel (A) shows that cognitive neuroscience and social care were the most frequent research areas, followed by clinical neurology and neuropsychology. Panel (B) indicates that most participants were at early career stages, predominantly graduates, PhD students, and postdoctoral researchers. Panel (C) displays the distribution by country, with Argentina and Brazil having the highest representation. Gender is disaggregated, showing a predominance of female participants across most countries.

A total of 130 participants completed the survey (out of 309 who started it and answered at least one question). Respondents’ ages averaged 40.25 years (± 12.18), 68.5% were female and 31.5% male, and 96 participants (73.8%) resided in LMICs and 34 (26.2%) in high‐income countries (HICs). The three most selected fields of work in dementia were cognitive neuroscience (16.2%), dementia/social care (13.7%), and clinical neurology (12.7%). Sociodemographic data, including gender, country of residence, field in dementia research, and career stage, are summarized in Figure 1.

#### 3.2.2 Leadership in dementia field: competencies and contextual barriers

According to the responses to the survey, the essential skills required for a leader in the field of dementia include the ability to establish strong collaborations and build multidisciplinary teams, which 56.1% of respondents considered fundamental. Additionally, a leader must possess strong communication skills to convey knowledge to diverse audiences, with 56.1% of respondents highlighting this competence as crucial. Close contact and involvement with patients and caregivers within the community are also important skills, as 38.4% of participants noted. The ability to influence local policy decisions was deemed essential by 38.4%, as effective leadership could help shape policies that benefit this population. Finally, a strategic mindset aimed at securing funding and managing institutional challenges was valued by 33%.

Scientific progress depends on robust structures and systems that foster opportunities for researchers, and 52.3% of respondents reported that the scientific system in their country was either underdeveloped or not prioritized as public policy. Furthermore, 64.6% indicated that access to training opportunities was limited or virtually inaccessible, and 80.7% stated that funding sources, including salaries, grants, travel scholarships, and other forms of support, were scarce or non‐existent for early‐career researchers in their region. Regarding accessing essential research equipment, such as neuroimaging, biomarker panels, psychophysiological measures, and neuropsychological assessment tools, 57.7% reported significant limitations, while 10% noted that these resources, though available, were not accessible for research purposes.

#### 3.2.3 Key areas of capacity building

The development of scientific ideas requires adequate time and resources. According to the survey, only 39.2% of respondents hold full‐time academic positions, and 13% dedicate all their professional time to academia. Additionally, 33.1% of respondents reported not having access to a dedicated physical space for scientific activities, such as laboratories, offices, or workspaces.

In terms of research resources, cognitive tests, magnetic resonance imaging (MRI), and electroencephalogram (EEG) equipment are the most accessible, while positron emission tomography (PET) scans and cerebrospinal fluid (CSF) collection are far less available. Human resources present another significant challenge: 54.6% of teams reported having no salaried staff (e.g., study coordinators, raters, interns, administrative assistants), and 35.1% indicated they lacked fellows (e.g., PhD or postdoctoral students) on their teams.

Funding is a critical enabler of scientific advancement, not only for project execution but also for acquiring specialized skills and for improving data analysis capabilities. Despite this, 48.5% of respondents reported never having received funding for academic training. Nevertheless, research teams often function as key hubs for training opportunities. Among the respondents, 61.5% reported receiving specialized dementia training, and 66.2% stated that their groups provided regular learning opportunities, including team meetings, seminars, and consultation spaces.

In line with these challenges, limited access to financial support for research remains a significant barrier. Survey results indicated that 65.4% of respondents had not received any grants or financial support for research in the last 5 years. As expected, access to this sort of funding varies considerably across career stages. While undergraduate and graduate students report minimal access to this type of funding, a significant proportion of individuals at more advanced stages, including postdoctoral researchers (59.1%), assistant professors (64.3%), associate professors (75%), and full professors (71.4%), indicated having received financial support. Despite most respondents reporting not receiving individual funding, 65.4% participated in funded projects during this period. Of these, 41.1% were funded by local grants, 25.8% by international grants, and 32.9% by both.

Concerning conference and publication support, 65.4% of respondents reported having received travel grants or financial support for attending local or international conferences in the past 5 years. When asked about funding sources for these events, 31.5% of respondents typically apply for international grants, 26.2% rely on funding from their own institution, 24.6% rely on local grants, and 17.7% reported using other sources. Additionally, 69.5% of respondents indicate a lack of economic resources to cover publication costs.

Regarding the translation of research into community actions, 73.1% of respondents thought their work had not been applied or translated in their community. Nevertheless, 52.3% reported that their research team regularly organized community‐oriented initiatives such as workshops, public talks, and courses. Among those who confirmed conducting these activities, contributions mainly involved programs promoting lifestyle changes, memory training workshops, and the dissemination of information through social media. Furthermore, 68.5% of respondents indicated that their groups did not engage in structured science communication activities.

**FIGURE 2:**
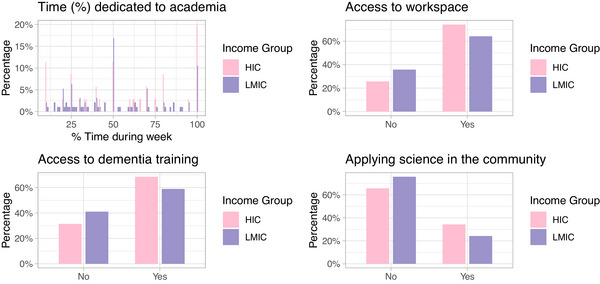
Overview of participants' academic engagement and access to research resources by income group (*n* = 130). Countries were categorized as high‐income countries (HICs) and low‐ and middle‐income countries (LMICs) according to the World Bank classification. (A) Distribution of percentage of time participants devote to academic work during a typical week. (B) Percentage of participants with and without access to dedicated workspace for research activities. (C) Percentage of participants reporting having access to dementia‐specific training. (D) Percentage of participants reporting applying scientific knowledge in community settings.

Figure 2 synthesizes the information presented. It summarises key aspects of the participants’ academic settings and access to research resources, including time dedicated to academic work, availability of a physical workspace, access to dementia‐specific training, and translation of scientific outputs for the community. These visual representations support the findings discussed in the previous section and highlight the diversity of experiences among researchers in the field of dementia.

## 4 DISCUSSION

### 4.1 Leadership and capacity‐building challenges

**FIGURE 3:**
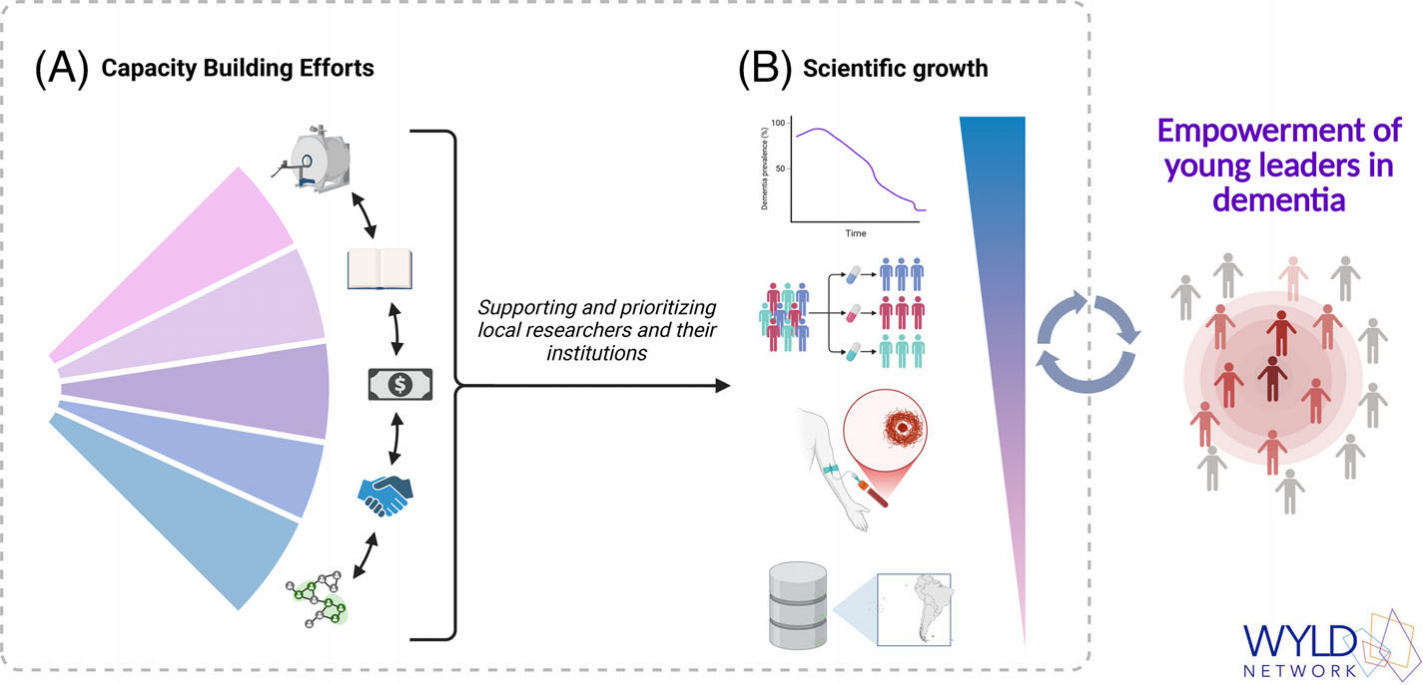
Diagram of process of capacity building and scientific growth promoted by WYLD network. (A) Capacity‐building efforts, including access to infrastructure, training, funding, networking opportunities, and local knowledge, aimed at supporting and empowering local researchers and their institutions. (B) Contribution of these efforts to scientific growth, as reflected in collaborative research, data generation, spatial analysis, and improved scientific access. Together, these processes create a continuous cycle that fosters the empowerment of young leaders in the field of dementia.

This article emphasizes the role of leadership skills in capacity building through the development of resources that support scientific advancement. Leaders act as agents who create opportunities and mobilize resources, contributing to the independence of scientific systems that remain heavily reliant on funding from a limited number of countries. As demonstrated by the results of our study and supported by numerous examples in the literature,^2^, ^3^, ^4^, ^5^, ^6^ scientific systems continue to be constrained by insufficient infrastructure, funding, and training. In a world where dementia cases are increasing significantly,^1^ the urgency of promoting and supporting individuals who can generate and translate scientific knowledge into effective public policies is critical^15^ (Figure 3).

In line with this perspective, the leadership representations emerging from the ISTAART Intermission reinforce the value of inclusive, collaborative, and communicative leadership styles, as well as the importance of having a strategic vision to overcome institutional and funding challenges. One key quality identified in the survey was the ability to establish collaborations with international teams. Whether international or local, collaboration can be conceptualized as a Vygotskian‐style Zone of Proximal Development,^16^ understood as the distance between the current level of development and the potential level that can be achieved through engagement with more experienced teams or those with complementary resources. Additionally, the ability to build multidisciplinary research networks emerged as a vital leadership trait. The integration of concepts and methodologies from a variety of disciplines can enrich research hypotheses, outcomes, and their broader impact. This quality must be supported by communication skills that enable the articulation and sharing of a common vision aligned with the team's goals.

While leadership plays a central role in advancing research capacity, it cannot operate in isolation. Its potential is often constrained by structural challenges. Numerous barriers hinder scientific development, most directly or indirectly linked to a lack of funding, particularly in LMICs.^2^ First, as mentioned, the lack of funding from scientific and governmental institutions is a significant obstacle. This situation reduces essential resources such as research assistants, statisticians, technological tools for data analysis, and suitable facilities for research activities. A third of researchers reported a lack of physical space (e.g., laboratory or office) to conduct their work. Additionally, more than half reported not having salaried staff (e.g., study coordinators, raters, administrative assistants), which forces researchers to take on tasks such as data entry and administrative coordination, reducing both the quantity and quality of time dedicated to creating new projects or even the analysis and the elaboration of their findings.

Furthermore, financial and logistical barriers hinder the possibility of publishing in certain journals due to the impossibility of covering publication costs. In this context, initiatives such as Research4Life play a crucial role, offering financial support to authors from eligible institutions in LMICs to publish in selected open‐access journals. These same barriers also limit participation in international scientific community events. Conferences provide a platform for dialogue and exchange, bringing together researchers worldwide and enabling connections, networking, and collaboration between teams. However, travel costs remain significant obstacles to scientific career advancement.^17^ In this regard, initiatives such as the Resources for LMICs from ISTAART contribute to addressing this issue by offering conference discounts, travel fellowships, and other forms of support. Additionally, hybrid‐format events such as AAIC Neuroscience Next help foster academic diversity and inclusivity by enabling broader participation across regions.^18^

The lack of funding for education and training is another issue frequently reported by researchers. Training translates into acquiring specific skills in the field, improved data analysis capabilities, and, consequently, disseminating this new knowledge to the rest of the team. More than half of our study's researchers reported never receiving funding for specialized training in dementia research. In parallel, most respondents indicated that their primary source of training has been within their own research teams, which have acted as small training hubs by organizing regular meetings, seminars, and consultation spaces. However, this is not a permanent, scalable, or formal solution to train the new generation of leaders in dementia research; effective and organized formal training is needed.

Beyond training limitations, time constraints emerged as a critical obstacle to research productivity. Only 39% of researchers hold full‐time positions, and just 13% dedicate all their time to academic pursuits. Several factors may contribute to this phenomenon. A key issue is that researchers’ salaries often fail to cover the cost of living in contexts where scientific systems are being dismantled. This financial insecurity forces many scientists to take on additional jobs to meet their basic needs, relegating research to after‐hours efforts. This situation profoundly impacts productivity, particularly for early‐career researchers, who face heightened challenges in establishing their academic careers. Without sufficient time and appropriate workspaces, productivity, measured in terms of knowledge generation and its potential applications, is significantly diminished.

Finally, more than 70% of researchers reported that their work had not been effectively translated into tangible benefits for the community. Compounding this issue is the lack of science communication activities. Approximately 70% of researchers reported that their group did not communicate results to the public.

Overcoming these barriers often requires more than individual efforts or international collaborations, which may not always be stable or long‐lasting. It calls for a strong and sustained commitment from local governments and political and economic decision makers, that is, a strategic investment in scientific and technological development. Within this context, leadership skills can play a pivotal role in capacity‐building processes.

A concrete example of this dynamic is the Latin American Initiative for Lifestyle Intervention to Prevent Cognitive Decline (LatAm‐FINGERS).^19^ This initiative represents the first randomized controlled trial in Latin America designed to prevent cognitive decline and dementia in 1200 participants across 13 centers in 12 LMICs, including Argentina, Bolivia, Brazil, Chile, Colombia, Costa Rica, Ecuador, Mexico, Peru, Puerto Rico, the Dominican Republic, and Uruguay. The structure of LatAm‐FINGERS was explicitly designed to train and support participating researchers by fostering leadership development at each center through targeted training strategies and the provision of infrastructure and equipment resources. In this way, the project aims not only to generate scientific evidence on interventions for dementia prevention, but also to consolidate a regional network of scientific leadership.

### 4.2 Building scientific leadership: A multilevel approach

Strengthening leadership within scientific environments requires coordinated efforts across three interconnected levels: individual, institutional, and societal.^14^ We propose a comprehensive approach that enhances researchers’ capacities, reinforces collaborative structures within research groups, and aligns public policy with long‐term scientific development.

At the individual level, leadership can be cultivated through targeted training in core competencies, including institutional and organizational management, effective organizational communication, and scientific writing.

At the institutional level, building leadership involves creating environments that enable distributed leadership, collaborative decision‐making, and interdisciplinary engagement. Key strategies include (1) fostering the integration of multiple disciplines within the same research group, (2) establishing strategic collaborations with research groups offering greater experience or complementary resources, facilitating mentorship and co‐leadership, (3) establishing regular and formal training programs within research groups, and (4) developing science communication projects to engage broader audiences and reinforce the visibility and societal value of science.

At the societal level, consolidating scientific leadership requires strong and sustained commitment from public institutions. Investments in science must transcend short‐term political agendas and be framed as a long‐term strategy to strengthen scientific and technological development, as well as national sovereignty.

### 4.3 The WYLD mission

WYLD is dedicated to empowering the next generation of dementia researchers through (1) training opportunities, (2) networking, and (3) collaboration with open data and open science initiatives. Its mission is to create a platform that enables researchers to advance their careers while contributing to the well‐being of people with dementia, their families, and their communities. The organization focuses on facilitating professional training and enabling connections between early‐career researchers and health institutions worldwide. Additionally, it supports researchers in attending international conferences and plays a key role in promoting brain health awareness and disseminating knowledge to the broader community.

### 4.4 Study limitations and future considerations

The study presented here has certain limitations that should be considered in future WYLD initiatives. The primary limitation, from which other issues stem, concerns the sample size. Future efforts should seek to include a larger and more geographically diverse group of participants, thereby enabling meaningful comparisons between researchers from LMICs and HICs. Also, there was both overrepresentation and underrepresentation of certain countries among the respondents, highlighting the need to further expand WYLD's global reach. In addition, substantial heterogeneity in the participants' career stages was observed. Increasing the sample size would enable a more detailed analysis of potential differences between early‐career researchers and more established investigators. A mismatch was observed in many respondents between their country of origin and their current country of residence. Future research should explore more deeply the role of migration in shaping research careers, as this appears to be a relevant but underexplored factor. Finally, regarding language accessibility, we acknowledge that the survey was only available in English at the time of dissemination. This may have limited participation for some potential respondents. We recognize this as a limitation and suggest that future studies offer the survey in multiple languages to improve accessibility and response rates.

## 5 CONCLUSIONS

Building a robust and globally equitable scientific community fundamentally requires a process similar to agriculture: Leadership skills must be developed in environments that offer real opportunities for growth. Our findings, based on the ISTAART Intermission at AAIC 2024 organized by WYLD and our survey of dementia researchers, highlight that in many contexts such opportunities remain a privilege rather than a standard practice for scientists, particularly in LMICs. It is about learning to foster and nurture: Prioritizing access to tools, protecting research time, and developing leadership skills are the next steps toward equitable and sustainable science.

In this context, initiatives led by professional societies and networks have become key drivers in supporting dementia researchers. Among them, WYLD stands out, serving as a global platform that empowers early‐career researchers through mentorship, training, and collaborative opportunities. These efforts are further strengthened through partnerships with other organizations such as ISTAART, which offers targeted mechanisms to support emerging researchers. Dedicated networking spaces such as the professional interest areas (PIAs), particularly initiatives like the PIA to Elevate Early Career Researchers (PEERs) or the Diversity and Disparities PIA, as well as efforts such as the ISTAART Ambassador Program, are actively cultivating the next generation of dementia researchers. These actions complement WYLD's mission and reinforce a global commitment to building a sustainable, inclusive, and diverse research community.

Equally essential are conferences and workshops, including major international gatherings like the AAIC, as well as regional events, that provide dedicated spaces for researchers to connect, establish collaborative networks, and engage in mutual learning. These encounters foster a stronger sense of community, encourage interdisciplinary exchange, and play a critical role in shaping a more inclusive and dynamic future for dementia research.

To conclude, the ISTAART Intermission session highlighted the spirit of collaboration and diversity among early‐career researchers who participated in its activities and discussions. Their collective engagement reflects the importance of creating supportive spaces for the development of future leaders in dementia research.

## CONFLICT OF INTEREST STATEMENT

F. Morello‐García, M. A. Hernández, E. Ruppert, and N. Dubey report support from the Alzheimer's Association to attend the AAIC. N. Corvalán reports support from the Alzheimer's Association to attend the AAIC and the AAIC Neuroscience Next. J. Llibre‐Guerra reports serving as Vice‐Chair of the World Young Leaders in Dementia (WYLD). M. Arruabarrena reports serving as Digital Manager of the WYLD, participation in the ISTAART Ambassador Program 2024‐2025, and support from the Alzheimer's Association to attend the AAIC and the AAIC Satellite Symposium. M. F. Clarens reports support from the Alzheimer's Association to attend the AAIC and the AAIC Advancements. G. Keller reports participation in the ISTAART Ambassador Program 2025–2026 and support from the Alzheimer's Association to attend the AAIC. C. S. Aguzzoli reports support from the Global Brain Health Institute, the Alzheimer's Society (GBHI ALZ UK‐23‐971089), the Alzheimer's Association (24AACSF‐1200375), and CAPES (88887.951210/2024‐00). R. Allegri reports support from the Alzheimer's Association (SG‐21‐715176‐LatAm‐FINGERS). C. Ardohain Cristalli reports support from the Alzheimer's Association to attend the AAIC Satellite Symposium. B. Bellaver reports support from the Alzheimer's Association (AARFD‐22‐974627), as well as additional support from the Alzheimer's Association to attend the AAIC. M. N. Best reports support from the National Institutes of Health (T32 NS007222) and the Alzheimer's Association (24AARFD‐1189285). I. Camargo Fontana and O. Ismail are full‐time employees of the Alzheimer's Association. K. Chen reports support from the National Institute on Aging (1K08AG084902) and the Alzheimer's Association (AACSF‐22‐970586). I. García‐Cordero reports support from the Holloway Family Fund of the Association for Frontotemporal Degeneration, the Bob Burros Family Memorial Fund of the American Brain Foundation and the American Academy of Neurology. S. Koerich reports support from the Alzheimer's Association (AARFD‐23‐1150704), as well as additional support from the Alzheimer's Association and the WYLD to attend the AAIC. M. C. López Moreno reports participation in the ISTAART Ambassador Program 2023‐2024, and support from the Alzheimer's Association to attend the AAIC and the AAIC Neuroscience Next. P. L. Ferreira reports support from the Alzheimer's Association (AARFD‐22‐974627). N. Magrath Guimet reports support from the Global Brain Health Institute, Alzheimer UK, and the Alzheimer's Association (GBHI ALZ UK‐22‐865809), as well as from Bluefields and UCSF. G. Povala reports support from the Alzheimer's Association (24AARFD‐1243899). A. Rocha reports support from the Alzheimer's Association (AARFD‐24‐1307665). M. S. Rodrigues reports support from the Alzheimer's Association (AARFD‐24‐1313939). M. S. Medeiros reports support from the Alzheimer's Association to attend the AAIC and from the Human Amyloid Imaging to attend HAI 2025. K. Seibert reports support from the Retirement Research Foundation and Eli Lilly. C. Sexton was a full‐time employee at the Alzheimer's Association at the time this work was performed. H. Wilks reports support from the National Institute on Aging (AG000030‐47). E. R. Zimmer has served on the scientific advisory board as a consultant or speaker for Nintx, Novo Nordisk, Biogen, Lilly, Magdalena Biosciences, and masima. He is also a co‐founder and minority shareholder of masima and reports support of CAPES (88881.996985/2024‐01),  CNPQ (435642/2018‐9, 312306/2021‐0, 409066/2022‐2, 447074/2023‐7, 409595/2023‐3,444880/2024‐0), Instituto Serrapilheira (R‐2401‐47242), FAPERGS (85053.824.30451.24062024), Alzheimer's Association (21‐850670, 22‐928689, 23‐1148735, BFECAA2024), National Academy of Neuropsychology (22‐92838), Michael J. Fox (MJFF‐ 023158), and  Ministério da Saúde/DECIT (00030420240118‐003490).  L. Crivelli reports serving as Chair of the WYLD and receiving support from the Alzheimer's Association (SG‐21‐715176‐LatAm‐FINGERS), the National Institutes of Health (3R01AG081394‐02S1), and the ADDI.

The remaining authors declare that they have no conflicts of interest to disclose. Author disclosures are available in the supporting information.

## FUNDING STATEMENT

None

## CONSENT STATEMENT

The study was approved by the Fleni Research Ethics Committee (registration code 14195), which waived the requirement for informed consent due to the characteristics of the survey.

## Supporting information



Supporting Information

Supporting Information
