# Supplementary material for: Capacity building in dementia research: insights from the World Young Leaders in Dementia
Source: Alzheimers Dement. 2025 Dec 17;21(12):e70667. doi: 10.1002/alz.70667 (PMC12710519; doi:10.1002/alz.70667)
Supplement: Supplementary file 2 — Supporting Information [file ALZ-21-e70667-s002.docx]

DEMOGRAPHICS

1. Age
2. Gender
3. Auto-refered ethnicity
4. Country of origin
5. Country of residence
6. Your profesión (e.g. psychologist, biologist, physician)
7. Your specialization (e.g. neuropsychologist, molecular biologist, neurologist)
8. Stage of career
9. Field of dementia you work

CAPACITY BUILDING

*Your opinion about opportunities in science*

(1) How developed is the research system for dementia in your country? Please consider public and private investment in science, infrastructure, salaries, and others.

(2) What do you see as the biggest barriers to developing leadership skills in dementia research in your region?

(3) How accessible is dementia-focused academic training for young researchers in your region? When responding, please consider training opportunities, team collaborations, and exchange scholarships.

(4) How accessible are local funding opportunities for young researchers in your region? When responding, please consider salaries, project grants, travel grants, and other funding opportunities.

(5) How accessible are equipment / technology devices for young researchers in your region? When responding, please consider neuroimaging and biomarkers equipment, psychophysiological measures tools, neuropsychological tools, and others.

(6) How accessible are qualified mentors and support networks for young dementia researchers?

(7) Do you think dementia research receives enough visibility and institutional recognition in your country? When responding, please consider public awareness campaigns, specific awards, recognition for young researchers, among other initiatives.

(8) What strategies would you propose to overcome language and cultural barriers in international collaboration in dementia research?

(9) What do you think are the key strengths that leaders in dementia research should possess?

(10) What are the primary benefits you've experienced from networking with other dementia researchers?

(11) How much of a priority do you think organizing community engagement events (e.g., workshops, public talks) should be for research leaders in the field of dementia?

(12) What challenges have you faced in organizing community engagement events?

(13) Researching in the field of dementia can be a process full of challenges.  What inspires you to commit to this work and drives you to keep going?

CAPACITY DATA

*Some data about your lab / team*

*This section focuses on data about your research team in terms of 5 key areas of capacity building: education, funding, infrastructure, collaboration with other teams, and community engagement.*

***Educational training***

(1) Have you received any specialized training in the dementia research field?

a. If yes, please specify the type of training: short courses, advanced or specialized programs lasting more than a year, rotations in specialized services, or other types.

(2) Does your research team have regular educational opportunities, such as weekly meetings or seminars?

(3) Have you ever received funding or support specifically for education or training purposes?

(4) In the past 5 years, have any team members participated in research stays or rotations?

1. If so, were these stays national or international?

(5) Are you currently mentoring or training other researchers?

***Funding opportunities***

(1) Do you have a full-time academic position?

(2) How much of your professional time is dedicated to academic work?

(3) Have you participated in any group projects funded by grants in the past 5 years?

a. If yes, were the project grants local, international, or both?

b. If not, has your team had experience applying for grants?

(4) Have you won any grants as a PI or co-director in the last 5 years?

a. If yes, were the project grants local, international, or both?

(5) In the past 5 years, have you or any team members received travel grants or financial support specifically for attending local or international conferences?

(6) To what sources of funding do you typically apply to attend scientific events?

(7) Do you have internal funding available for publication expenses?

***Infrastructure***

(1) Do you have access to a dedicated lab, office, or workspace for your research activities?

(2) What types of equipment does your research team have access to?

(3) Do you have any salaried administrative or study personnel on your team? (e.g. study coordinators, raters, interns, administrative assistant)

(4) How many research assistants are currently in your research team?

(5) How many fellows are currently in your research team? (e.g. PhD / postdoctoral students)

***Collaborations with other teams***

(1) Have you conducted studies in collaboration with any national team in the last 5 years?

(2) Have you conducted studies in collaboration with any international team in the last 5 years?

(3) Do international researchers collaborate on projects with your team as mentors?

***Community Engagement***

(1) Has your dementia research project been successfully translated or applied to the community in any way?

a. If you answered "Yes", please describe how.

(2) Does your research team regularly organize community-oriented events (e.g., workshops, courses, public talks)?

(3) Does your team have a dedicated science communication or outreach group?

(4) Which platforms do you use to disseminate your research findings?
